# Acknowledgment to Reviewers of *European Journal of Investigation in Health, Psychology and Education* in 2020

**DOI:** 10.3390/ejihpe11010009

**Published:** 2021-01-28

**Authors:** EJIHPE Editorial Office

**Affiliations:** MDPI AG, St. Alban-Anlage 66, 4052 Basel, Switzerland

Peer review is the driving force of journal development, and reviewers are gatekeepers who ensure that *European Journal of Investigation in Health, Psychology and Education* maintains its standards for the high quality of its published papers. Thanks to the cooperation of our reviewers, in 2020, the median time to first decision was 16 days and the median time to publication was 42.5 days. The editors would like to express their sincere gratitude to the following reviewers for their precious time and dedication, regardless of whether the papers were finally published:
Abuin, VanesaLee, Chang WonAlbendín-García, LuisLemberger-Truelove, MatthewAlbornoz-Cabello, ManuelLerma, ClaudiaAl-Imam, AhmedLesiuk, TeresaAlmeida, LeandroLewko, JolantaAlonso, MartaLi, ManyuAlqraini, FaislLi, WeijuanAmer Jid Almahri, FatimaLiu, ChangqiAnastasiou, SophiaLópez, DanielArensberg, Mary BethMagnavita, NicolaArmaou, MariaMahan, MichaelAsif, MuhammadMalinauskas, RomualdasBaldwin, PaulaMaraffi, SabinaBargiel-Matusiewicz, KamillaMartínez Ramón, Juan PedroBarneva, RenetaMartínez-Rodriguez, AlejandroBartczuk, RafałMcCullough, LauraBeard, LarryMcKay, NaomiBergold, SebastianMd-Yunus, Sham’AhBrough, LouiseMedawar, EvelynBuksnyte–Marmiene, LoretaMenin, DamianoBuonomo, IlariaMenozzi, DavideBüssing, Alexander GeorgMesenge, ChristianCalado, FilipaMétioui, AbdeljalilCalvete, EstherMiranda, JonatanCapone, VincenzaMizuno, KohCarmassi, ClaudiaMontaña Blasco, MireiaChang, YunjeongMugabi, IvanChoate, PeterMurphy, PhillipCodina, NuriaMynampati Arunadithya, Bharani KrishnaColnar, SimonNagy, NoémiCornelissen, PiersNapal, MaríaCortés-Rodríguez, Alda ElenaNash, PoppyDa Silva Souza, Ivan DomicioNavarro, RaulDal Mas, FrancescaNavarro-Pérez, CarmenDe La Fuente, JesúsNeocleous, GregoryDe La Torre-Montero, Julio-CésarNkuba, MabulaDe Las Heras-Pedrosa, CarlosNovak, MirandaDempsey, HeidiOkuda, MasayukiDereń, KatarzynaOuellette, RachelDi Giacomo, DinaParv, LuminitaDlouha, JanaPaschke, KerstinDohi, ToshifumiPavliuk, RomanDombrovskis, ValerisPellas, NikolaosDonato, LuigiPereira De Figueiredo, InêsDore, BettyPerez, FranciscoDriessen, GeertPérez-Bermejo, MarcelinoDuradoni, MirkoPerlini, CinziaDziechciarz, JózefPino-Juste, MargaritaEdvardsson, Ingi RunarPizarro, JoseEhrsson, Ylva TiblomPlatania, ChiaraEl Ghoch, MarwanPower, ThomasElhady, MarwaPragai Olswang, GillieFeng, YaohuaPrieto, LeonelFernandez Salinero, SamuelPyżalski, JacekFerreira, Maria EduardaQuevedo-Blasco, RaúlGerke, OkeRenoult, LouisGómez Carrasco, Cosme JesúsRodríguez, FranciscoGreco, GianpieroRyabov, IgorGuan, ShanshanRzońca, PatrykGümüş, SedatSáez Padilla, JesúsGutiérrez Ortega, MónicaSalvador-García, CelinaHansmann, RalphSchumm, WalterHarrison, DavidSeifried-Dübon, TanjaHayward, Richard DavidSilvia, PaulHerrenkohl, ToddSobba, KristenHerrera-Peco, IvánSousa, Maria JoséHubáčková, ŠárkaStuchlikova, IvaHudomiet, PéterSulla, FrancescoHulme, JulieSzczerbinski, LukaszHynes, KristenTamez, MarthaIanos, Maria GratielaTrigueros, RubénIbáñez-González, MaríaTsikouras, PanagiotisI-Shiang, TzengVan Hoof, ChrisIzydorczyk, BernardettaVanduhe, Vanye ZiraJones, JohnVarelas, VassileiosJukić Peladić, NikolinaVaughn, AmberKantono, KevinVittori, AlessandroKatsantonis, IoannisVoroshilova, AnzhelikaKauffman, JamesWalker-Gleaves, CarolineKeinonen, TuulaWang, Qian JaniceKelly, KimberlyWard, NicolaKelly, LouiseWinter, DavidKevorkian, MelineWojciechowski, WallyKidger, JudiWoo, JenniferKiviruusu, OlliYager, ZaliKlímová, BlankaYagi, TadashiKlizienė, IrinaYazykova, ElenaLachmann, BerndYoung, ChaseLangos, ColetteZhu, Bo-Wei

